# PADI1 and Its Co-Expressed Gene Signature Unveil Colorectal Cancer Prognosis and Immunotherapy Efficacy

**DOI:** 10.1155/2022/8394816

**Published:** 2022-11-26

**Authors:** Yi-ran Zhang, Lei Zhang, Feng Li, Jia-shuai He, Jin-feng Xuan, Cong-cong Chen, Chao Gong, Yun-long Pan

**Affiliations:** ^1^Department of Gastrointestinal Surgery, The First Affiliated Hospital of Jinan University, Guangzhou 510632, China; ^2^Department of General Surgery, Wuzhou Red Cross Hospital, Wuzhou, Guangxi 543000, China

## Abstract

Peptidyl arginine deiminase 1 (PADI1) catalyzes protein citrullination and has a role in regulating immune responses. The tumor immune microenvironment has been reported to be important in colorectal cancer (CRC), which was correlated with the ability of CRC patients to benefit from immunotherapy. However, there is a lack of molecular markers for matching CRC immunotherapy. Previously, single-gene risk models have only considered the effect of individual genes on intrinsic tumor properties, ignoring the role of genes and their co-expressed genes as a whole. In this study, we analyzed the differential expression of PADI1 in colorectal cancer (CRC). We found that PADI1 was highly expressed in CRC. Subgroup survival analysis revealed a prognostic survival difference for PADI1 in CRC patients aged less than 65 years, male, *T* stage, N0, M0, and stage I-II (*p* < 0.05). In addition, we analyzed the functions and signaling pathways associated with PADI1 in CRC and found that it was highly enriched in several immune-related functions and pathways. Then, a set of PADI1 co-expressed genes (PCGs) risk-prognosis scores was developed with PADI1 as the core, which could accurately predict the prognosis of CRC (*p* < 0.05). PCGs risk score can be an independent prognostic factor for CRC. A new set of Norman plot models were developed for clinical characteristics with age, sex, and TNM stage, which can accurately predict CRC 1, 3, and 5 years survival, and calibration curves and decision curve analysis (DCA) validated the accuracy of the models. The risk score assessed the immune microenvironment of CRC and found that the immune score was higher in the low-risk group, and CD4+ T cells, helper T cells, and eosinophils were more infiltrated in the low-risk group (*p* < 0.05). Immunotherapy efficacy was better in the low-risk group (*p* < 0.05). The underlying mechanism may be that the high-risk group of PCGs was enriched in some pathways that promote immune escape and immune dysfunction. In conclusion, PCGs may better predict CRC prognosis and immunotherapeutic response.

## 1. Introduction

Colorectal cancer (CRC) is the second leading cause of death from cancer worldwide, accounting for approximately 10% of all cancer diagnoses and cancer-related deaths worldwide [1]. Its age of onset is advancing each year [2]. Colorectal cancer can usually be cured by surgery in the early stages of the disease, with a 5 years relative survival rate of about 90% [3]. Still, once colorectal cancer progresses to the middle and late stages, its 5 years survival rate is less than 50%, and the key to a good prognosis is early diagnosis [4]. Once colorectal cancer is diagnosed, the preferred treatment is still surgical resection and a postoperative combination of chemotherapy, radiotherapy, antiangiogenic therapy, immunotherapy, etc., but drug resistance still inevitably occurs [5–8]. Therefore, it is essential to understand the mechanism of colorectal cancer, find new tumor markers, and develop new targeted drugs to detect colorectal cancer more accurately, which has important research prospects and clinical significance.

PADI1, peptidyl arginine deiminase 1, is a calcium-dependent cysteine hydrolase that can mediate the citrullination of post-translational proteins [9]. It is a member of the PADs family and its primary function is to catalyze the conversion of arginine residues to citrulline residues in post-translational proteins [10]. A close association with the progression of oral mucosal cancer, breast cancer, and pancreatic ductal carcinoma has been reported in several publications but not in colorectal cancer [11–13]. In the subsequent studies, where genes regulate cellular traits in the form of networks, single-gene studies do not reveal the intrinsic properties of cancer more accurately, and a system consisting of a combination of the target gene and its co-expressed genes is required better to predict the biological properties of tumors and survival prognosis [14, 15].

In this study, we first investigated the expression of PADI1 in CRC and its impact on CRC survival prognosis and analyzed the function and enriched signaling pathways of PADI1 in CRC. The PADI1-relatedco-expression gene network was mapped, and a risk-prognosis model was developed. By using this model, differences in immune microenvironment status of CRC and differences in the efficacy of immunotherapy could be accurately predicted.

## 2. Materials and Methods

### 2.1. Pan-Cancer Expression and Survival Analysis of PADI1

TIMER is a web tool created by Harvard University's Professor of Immunoinformatics, TIMER (Tumor Immune Estimation Resource), at https://cistrome.shinyapps.io/timer/. UALCAN is a comprehensive, user-friendly, and interactive web resource for analyzing cancer OMICS data. We entered the gene name “PADI1” in UACALN and selected “COAD” and “READ” for tumor type to observe the expression of PADI1 in colorectal cancer. The difference in PADI1 expression in colorectal cancer was observed.

### 2.2. Analysis of Gene Ontology and Kyoto Encyclopedia of Genes and Gene Sets

We performed the differential analysis of the PADI1 high and low expression groups defined in the previous PADI1 survival analysis by selecting |logFC| > 0.585, *p* < 0.05, to obtain differential genes, including 69 upregulated genes and 128 downregulated genes. The molecular function (MF), biological process (BP), and cytological components (CC) of the differential genes were analyzed using the “clusterProfiler” in the R package. Components (CC) and KEGG pathway analysis.

### 2.3. Gene Co-Expression Analysis

Enter the search term “PADI1” in “Protein Name” on the STRING website, and select “*Homo sapiens*” for “Organization.” A total of 200 proteins interacting with PADI1 were identified. By using Cytoscape software, we analyzed the protein interactions with different color lines and formed a visual graph.

### 2.4. Construction and Validation of PCGs Risk Model

Three independent co-expressed genes were integrated, resulting in 237 co-expressed genes. After univariate Cox regression analysis, we used the R package “caret” to open a prognostic model for colorectal cancer with ten gene signatures by least absolute shrinkage and selection operator (LASSO) regression, and the formula of the risk score model was established as follows: risk score = ∑(*β*i ^*∗*^ Expi). *i* index represented the significant prognostic genes analyzed by Lasso regression. *β*i represents the beta coefficient of these genes.

### 2.5. Plotting of Norman Diagrams

Cox univariate and multivariate regression analysis of PCGs was performed with the “survival” R package. Age, sex, PCGs, and clinical stage were included as variables, and the prognostic model was constructed by using the R package “rms.”

### 2.6. The Relationship between the Construction of the CRC Immune Landscape and Risk Scores

Bioinformatics analysis based on transcriptomic data, several reliable algorithms have been established to quantify the relative proportions of immune swelling cells in individual samples, including ESTIMATE and cell classification methods [16]. In the present study, we calculated and compared the content of immune swelling cells between the high-risk and low-risk groups. Briefly, estimation allowed us to calculate the immune score and estimated score for each patient. We also assessed the correlation between risk score and immune swelling cells using Spearman's correlation analysis. Finally, we measured the CIBERSORT to measure 22 immune cells in tissues, including seven T cell types, naive B cells and memory B cells, plasma cells, NK cells, and myeloid subpopulations [17].

### 2.7. The Relationship between Risk Score and Immunotherapy

The TCIA database was developed mainly based on the next-generation sequencing data from TCGA. Each patient is analyzed separately. ID, disease, gender, and age information, we focus on the information in the IPS column. The IPS (immunophenoscore) column has four items with different attributes that can be good predictors of CTLA-4 and PD-1 responsiveness [18]. The tumor immune dysfunction and exclusion (TIDE) algorithm models other tumor immune escape mechanisms, including tumor-infiltrating cytotoxic T lymphocyte (CTL) dysfunction and immunosuppressive factor exclusion of CTL [19]. A higher TIDE score indicates that tumor cells are more likely to induce immune escape, thus indicating a lower response rate to ICI therapy.

### 2.8. Gene Set Enrichment Analysis

Gene set enrichment analysis (GSEA) was performed on risk genes to obtain this model's HALLMARK and KEGG pathways for MsigDB (c2.cp.kegg.v7.5.1.symbols.GMT; h.all.v7.5.1.symbols.GMT). The genes expressed between the high- and low-risk categories were studied for gene set enrichment. The alignment of this gene set was tested 1000 times to demonstrate its ability to sustain function.

## 3. Results

### 3.1. PADI1 Expression Levels and Survival Prognosis in CRC

**Figure 1:**
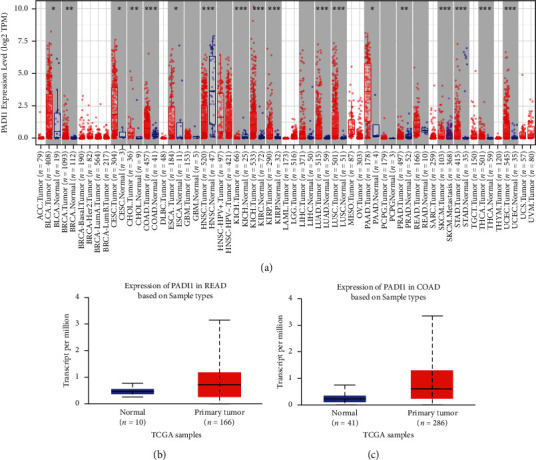
(a) Differential expression of PADI1 in pan-cancer; (b) PADI1 expression was higher in rectal cancer tissues than in normal tissues; and (c) PADI1 expression was higher in colon cancer than in normal tissues.

**Figure 2:**
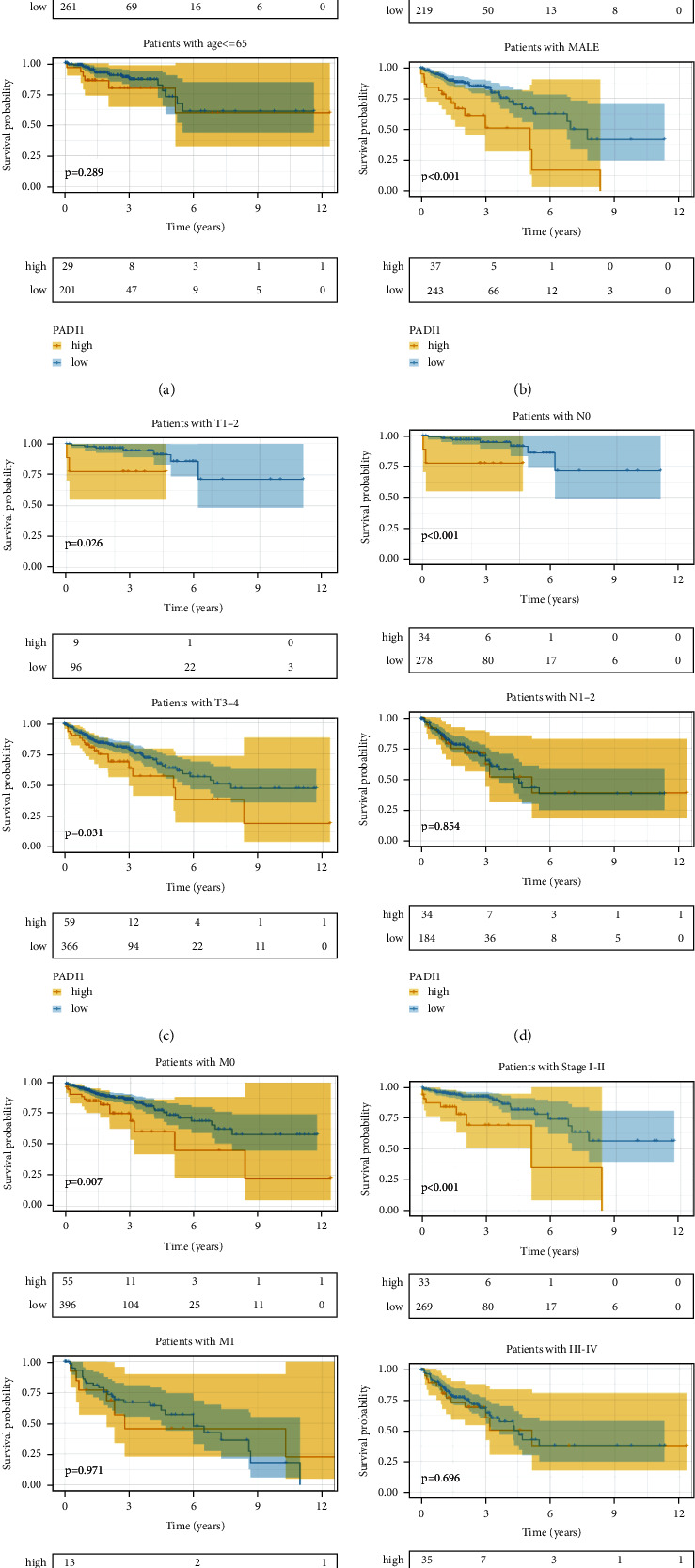
Survival analysis of PADI1 in diverse clinical subgroups of CRC. (a) Survival analysis of PADI1 in CRC in the age subgroup; (b) survival analysis of PADI1 in CRC in the gender subgroup; (c) survival analysis of PADI1 in CRC in the tumor size subgroup; (d) survival analysis of PADI1 in CRC in the lymph node metastasis subgroup; (e) survival analysis of PADI1 in CRC in the distant metastasis subgroup; and (f) survival analysis of PADI1 in CRC in TNM staging subgroup.

We first observed the difference in mRNA expression levels of PADI1 in pan-cancer, and the TIMER database showed that PADI1 was expressed at high and at low to medium levels (Figure 1(a)). Next, we looked at the expression of PADI1 in COAD and READ on the UACLAN website, respectively. The results showed that PADI1 expression was higher in cancer tissues than in paraneoplastic tissues in COAD and READ (*p* < 0.05, Figures 1(b)–1(c)). To further clarify the survival prognosis differences of PADI1 in different clinical features of CRC, we observed the survival differences of PADI1 by six clinical features, namely, age, gender, tumor size, lymph node metastasis, distant metastasis, and TNM stage, respectively (Figure 2).

### 3.2. GO and KEGG Analysis of PADI1

**Figure 3:**
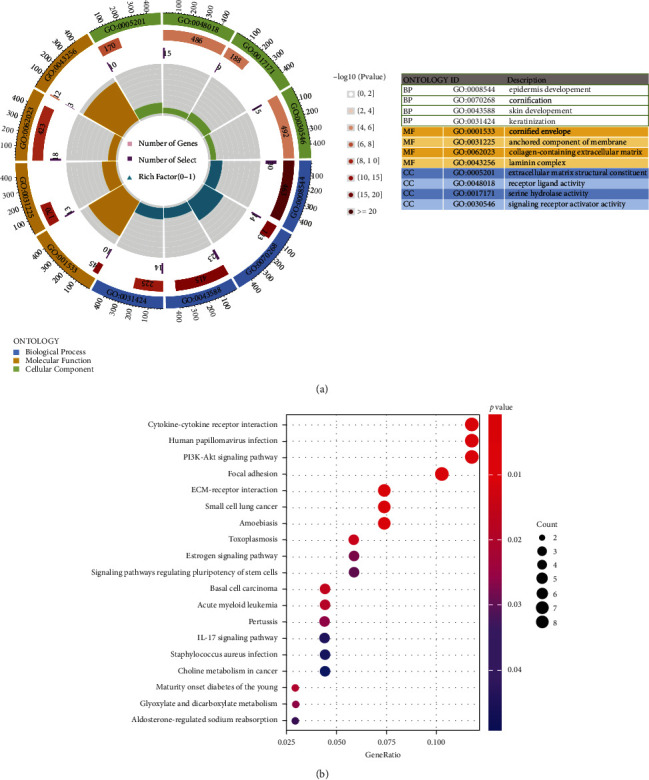
GO and KEGG analysis of PADI in colorectal cancer. (a) GO analysis of PADI1 in colorectal cancer; (b) KEGG analysis of PADI1 in colorectal cancer.

There were 96 differential genes in the PADI1 high and low expression groups (|logFC| > 0.5, *p* < 0.05), including 80 upregulated genes and 80 downregulated genes. GO analysis showed that differential genes were mainly enriched in BP for functions such as epithelial cell differentiation and keratinization, CC for extracellular matrix remodeling and signaling receptor activation, and MF for cell membrane fixation components (Figure 3(a)). KEGG pathway analysis showed that the differential genes were enriched in several functions that promote intercellular interactions, extracellular matrix receptor interactions, and PI3K-AKT signaling pathway (Figure 3(b)).

### 3.3. Co-Expression Network Construction of PADI1

**Figure 4:**
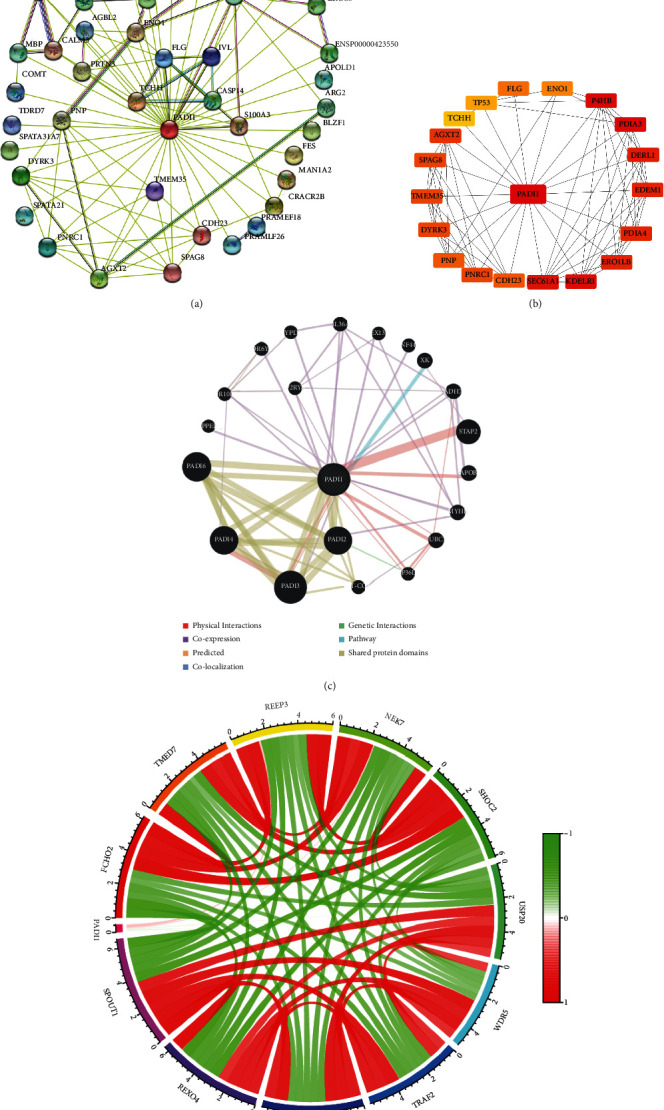
Protein-protein interactions analysis of PADI1. (a) PADI1 co-expression network constructed by STRING; (b) PADI1 protein-interaction network constructed by Cytoscape; (c) PAID1 protein-interaction network constructed by GenneMANIA website; and (d) PADI1 co-expression network constructed by TCGA database.

The top 50 genes co-expressed with PADI1 were selected (Figure 4(a)). Next, the top 20 genes that interacted most closely with PADI1 were further mapped using Cytoscape software (Figure 4(b)). The GenneMANIA website further predicted the specific interactions of PADI1 with some genes with physical binding, co-expression, etc., (Figure 4(c)). Finally, we used the colorectal cancer microarray in the TCGA database to map 11 genes in which PADI1 was associated (*p* < 0.05, Figure 4(d)). The abovementioned results reveal the core value of PADI1 in the cancer regulatory network.

### 3.4. Construction and Validation of a Risk Score for PADI1 Co-expression Genes

**Figure 5:**
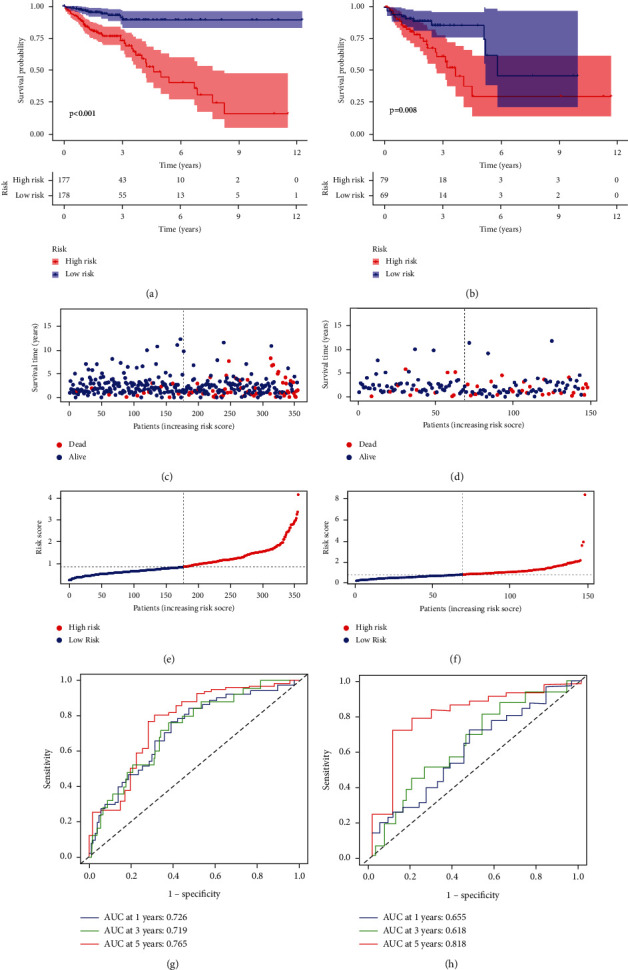
Survival analysis and prognostic performance of PCGs signature in colorectal cancer. (a) Kaplan–Meier analysis for overall survival in colorectal cancer between high- and low-risk patients in the training set (log-rank test, *p* < 0.001). (b) Kaplan–Meier analysis for overall survival in colorectal cancer between high- and low-risk patients in the Kaplan–Meier analysis for overall survival in colorectal cancer between high- and low-risk patients in the internal validation set (log-rank test, *p* < 0.001). (c) PCGs survival status in the training cohort of colorectal cancer patients. (d) PCGs survival status in the internal validation cohort of colorectal cancer patients. (e) PCGs risk scores in the training cohort of colorectal cancer patients. (e) PCGs risk scores in the internal validation cohort of colorectal cancer patients. (f) Receiver operating characteristic (ROC) curve survival at 1/3/5 years in the training set predicted by PCGs. (g) Receiver operating characteristic (ROC) curve survival at 1/3/5 years in the internal validation set predicted by PCGs. (h).

We conducted a univariate Cox regression analysis using a total of 30 genes co-expressed with CRC prognosis. Subsequently, we extracted 13 PCGs obtained from Lasso regression analysis. A prognostic model of CRC risk with PADI1-associated gene (PCGs) signature was constructed, and the CRC cohort in TCGA was randomly divided into two cohorts in the ratio of 7 : 3, the training cohort and the internal validation cohort, and GSE39582 was used as the external validation cohort. In both the training cohort and the internal validation cohort, a higher proportion of high-risk patients died, while a higher proportion of low-risk patients survived long-term (*p* < 0.05, Figures 5(a)-5(b)). As shown in Figures 5(c)–5(f). These samples were risk scored and ranked to determine whether expression levels varied systematically with a risk score. The results of the ROC curves are shown in Figures 5(f)-5(g), with prognostic predicted AUC of 0.726, 0.719, and 0.765 for the training cohort at 1, 3, and 5 years, respectively; and predicted AUCs of 0.655, 0.618, and 0.818 (Figures 5(g)-5(h)), indicating that the model has a good predictive effect. The established prognostic features were then further validated using GSE39582 as an external validation cohort. The results showed that the Kaplan–Meier survival curve showed a worse prognosis in the high-risk group than in the low-risk group (*p* < 0.05, Supplementary Figure 3A), and the proportion of risk varied with increasing risk score (Supplementary Figure 3B-3C), and the area under the ROC curve showed AUC of 0.628, 0.661, and 0.648 at 1, 3, and 5 years, respectively (Supplementary Figure 3D).

### 3.5. Establishment of Norman Diagrams for PCGs

**Figure 6:**
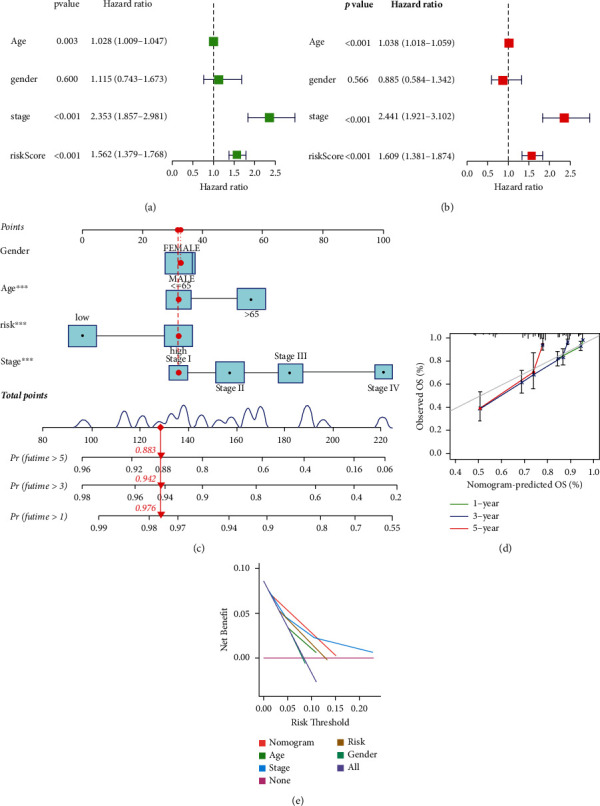
Construction of PCGs nomogram. (a) Univariate Cox regression revealed that the PCGs was an independent prognostic predictor of overall survival in the TCGA dataset. (b) Multivariate Cox regression revealed that the PCGs were an independent prognostic predictor of overall survival in the TCGA dataset. (c) Nomogram based on age, gender, clinical stage, and PCGs. (d) Calibration plots of the nomogram for the prediction of overall survival at 1, 3, and 5 years in the TCGA dataset. (e) Decision curve analysis (DCA) in the TCGA dataset. ^*∗∗∗*^*p* < 0.001.

To validate these candidate prognostic genes as independent biomarkers, univariate and multivariate Cox regression analyses were used to assess whether the predicted value was an independent prognostic factor (Figures 6(a)-6(b)). The risk scores of age and stage combined were selected to construct the Nomogram model, as shown in Figure 6(c). The corrected plots of the Nomogram (Figure 6(d)) show better agreement between the predicted OS results and the actual observations, indicating the good predictive performance of the PCGs prognostic model. The results show that age, gender, stage, and risk score prognostic characteristics incorporated in the model.

### 3.6. PCGs Were Highly Expressed in the Immune Microenvironment

**Figure 7:**
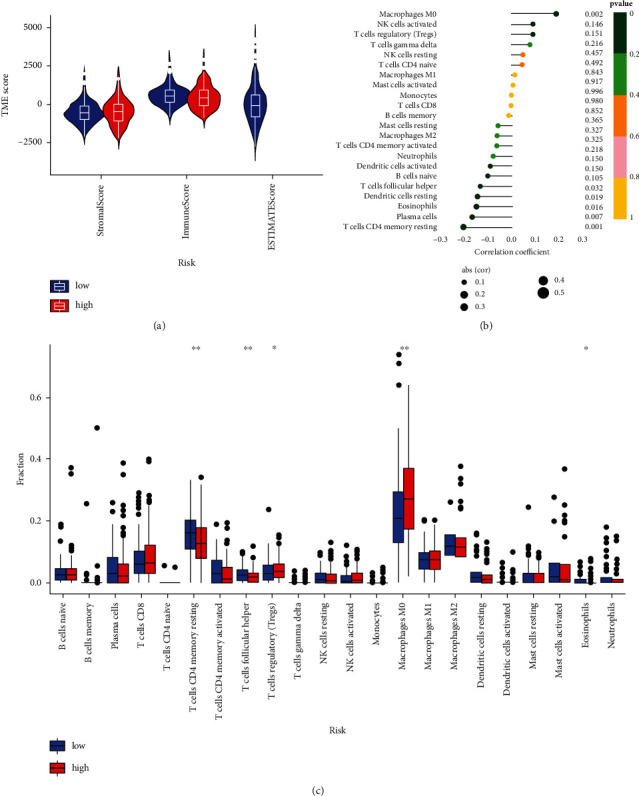
Correlation between PCGs risk score and immune microenvironment. (a) Patients with colorectal cancer with high-risk scores had higher immune scores, stromal scores, and ESTIMATE scores than those with low-risk scores. (b) Correlation of PCGs risk scores with immune cells. Circles represent correlation levels. (c) Comparison of infiltrating cell abundance in high- and low-risk subgroups. The upper and lower ends of the boxes represented the interquartile range of values. The line in the box represented the median and the dots indicated outliers. ^*∗*^*p* < 0.05, ^*∗∗*^*p* < 0.01.

Immune scores were higher in the low-risk group than in the high-risk group, with no difference in stromal scores (Figure 7(a)). There was a positive correlation with M0 macrophages and a negative correlation with CD4+ T cells, plasma cells, eosinophils, dendritic cells, and helper T cells (Figure 7(b)). CD4+ T cells were more abundantly infiltrated in the low-expression group, and Treg cells and M0 macrophages were more abundantly implanted in the high-risk group (Figure 7(c)).

### 3.7. Correlation of PCGs with Immune Checkpoints and Chemokines

**Figure 8:**
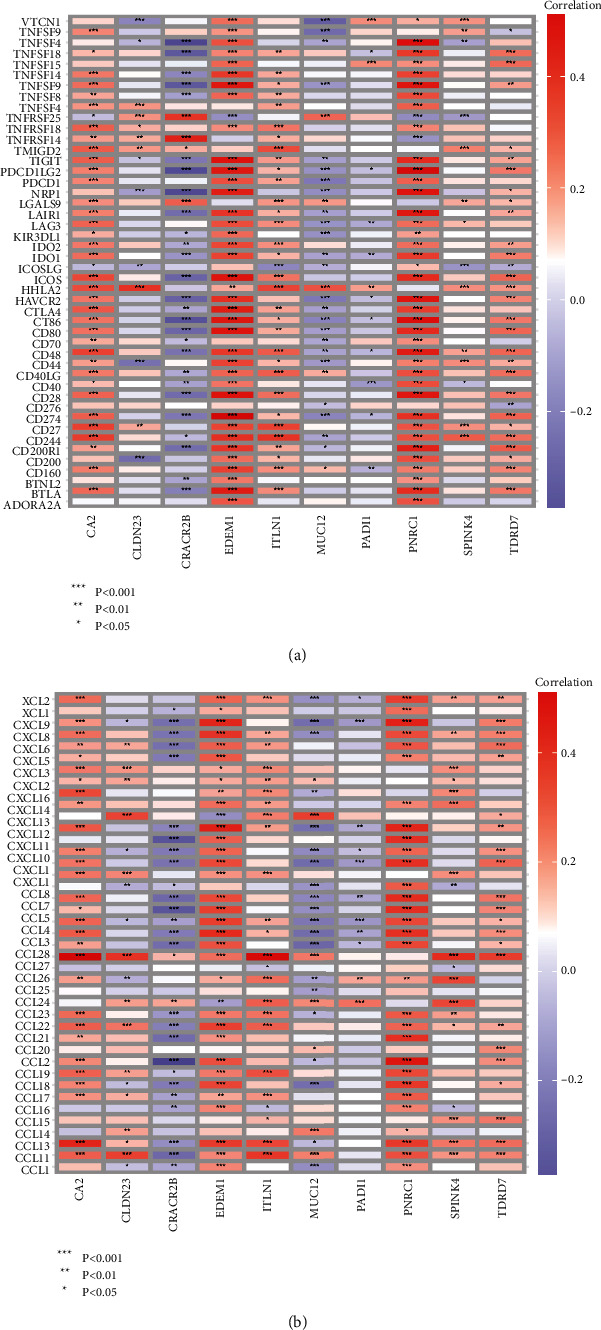
Association of 10 genes in PCGs with immune-related genes. (a) Correlation of immune checkpoints with 10 genes in PCGs. (b) Correlation of chemokines with 10 genes in PCGs. ^*∗*^*p* < 0.05, ^*∗∗*^*p* < 0.01, ^*∗∗∗*^*p* < 0.001.

To clarify the correlation of genes in PCGs with immune checkpoints, we correlated PADI1 and co-expressed genes with 47 immune checkpoints and 42 chemokines. The results were presented as heat maps, which showed that PADI1, MUC12 and CRACR2B were negatively correlated with immune checkpoints overall, CA2, CLDN23, EDEM1 ITLN1, PNRC1, SPINK4 and TDRD7 were positively correlated with immune checkpoints (Figure 8(a)); PADI1, MUC12 and CRACR2B were negatively correlated with chemokines in general, and CA2, CLDN23, EDEM1, ITLN1, PNRC1, SPINK4 and TDRD7 were positively correlated with chemokines in general (Figure 8(b)).

### 3.8. Relationship between PCGs Risk Scores and Immunotherapy

**Figure 9:**
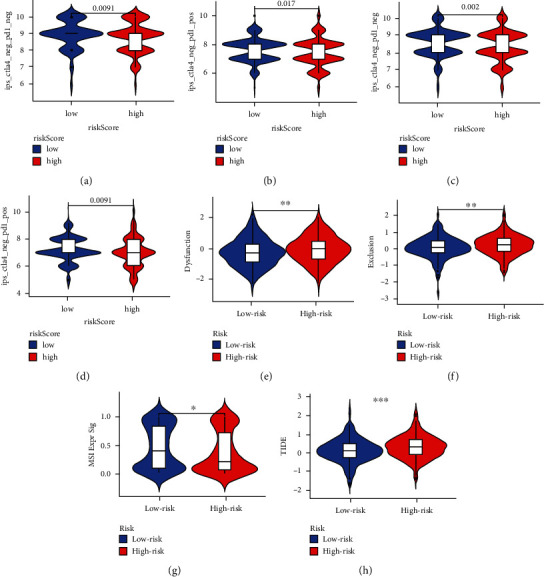
The PCGs risk score predicted immunotherapeutic benefits. (a–d) The relative distribution of IPS was compared between PCGs scored high versus low groups in TCGA-CRC. (e) Distribution of immune dysfunction in high- and low-risk groups of PCGs. (f) Distribution of immune exclusion in high- and low-risk groups of PCGs. (g) Distribution of microsatellite instability (MSI) in high- and low-risk groups of PCGs. (h) Distribution of TIDE score in high- and low-risk groups of PCGs. ^*∗*^*p* < 0.05, ^*∗∗*^*p* < 0.01, ^*∗∗∗*^*p* < 0.001.

Next, we further analyzed the relationship between IPS scores and risk scores, and Figures 9(a)–9(d) show that the overall IPS scores were higher in the low-risk group than in the high-risk group (*p* < 0.05, Figures 9(a)–9(d)). The TIDE algorithm assessed the immunodeficiency, immune escape, MSI and TIDE scores of CRC, and the results showed that the immunodeficiency and immune escape ability of the low-risk group were lower than those of the high-risk group; The MSI level was higher than that of the high-risk group; and the TIDE score was lower than that of the low-risk group *p* < 0.05, Figures 9(e)–9(h)). The abovementioned results indicated that the immunotherapy efficacy was better in the low-risk group than in the high-risk group in the risk score constructed by PCGs.

### 3.9. Identification of PCGs Risk Scores for Functional Enrichment Analysis

**Figure 10:**
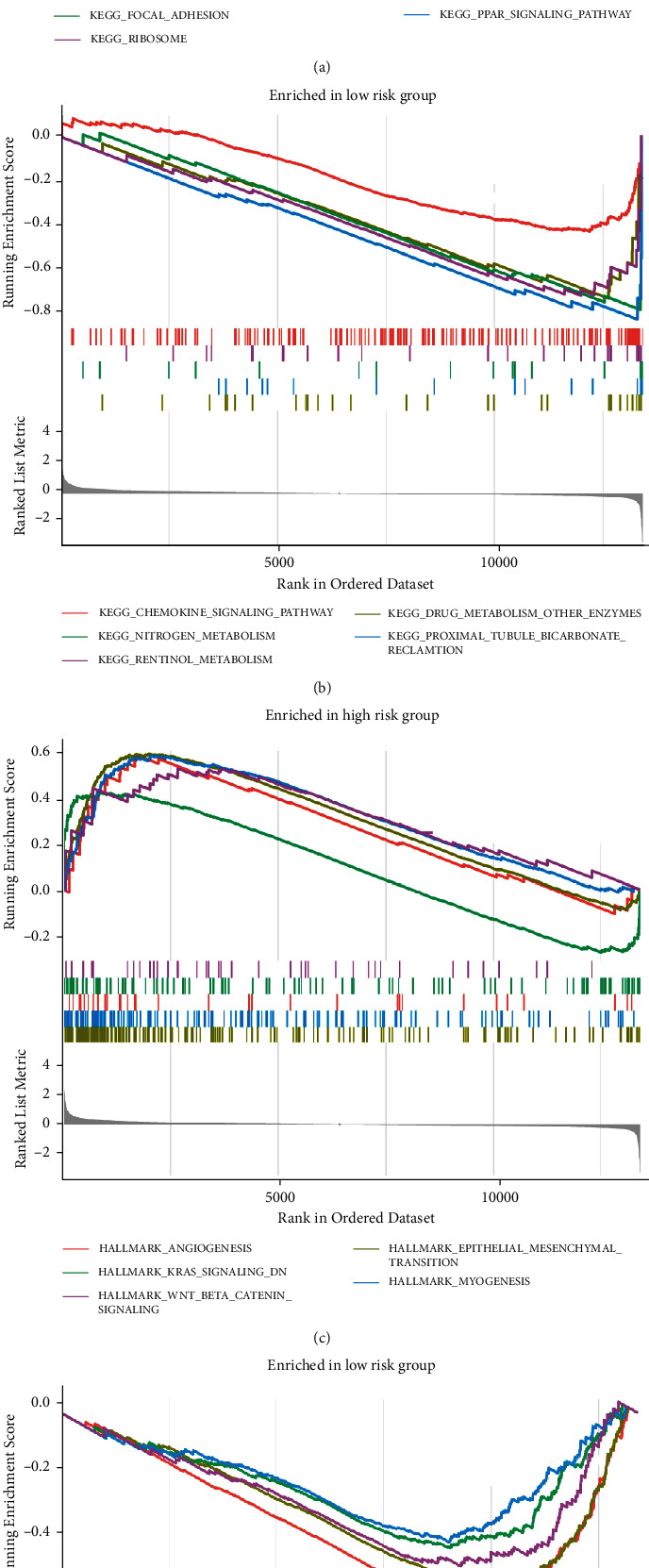
GSEA of high- and low-risk samples based on the prognostic index of PCGs signature. (a) Enriched genomes in the KEGG set for high-risk samples. Each line represents a specific genome in a unique color, with upregulated genes located to the left near the origin of the coordinates, compared to downregulated genes located to the right of the *x*-axis. Only genomes with NOM *p* < 0.05 and FDR *q* < 0.05 were considered significant, and only the five leading genomes are shown in the figure. (b) Genomes enriched in KEGG with low-risk group samples, and only 5 leading genomes are shown in the figure. Only 5 leading genomes are shown in the figure. (c) Gene sets are enriched in the HALLMARK collection by high-risk samples. Only the 5 leading genomes are shown in the figure. (d) Gene sets are enriched by low-risk samples in the HALLMARK set. Only the 5 leading genomes are shown in the figure.

GSEA was employed to identify the pathways enriched in the HALLMARK and KEGG databases, showing the top five pathways in the NES score. The top five signaling pathways in the high-risk group in the KEGG database were mainly pathways for intercellular interactions, and the low-risk group was mainly enriched in some molecular metabolic pathways (Figures 10(a)–10(b)); the top five pathways in the high-risk group in the HALLMARK database were the top five pathways in the high-risk group in the HALLMARK database were angiogenesis, epithelial-mesenchymal transition, KRAS, and WNT signaling pathways; the low-risk group was enriched in cell cycle molecules and oxidative phosphorylation pathways (Figures 10(c)–10(d)).

## 4. Discussion

There is growing evidence linking the PADs family to carcinogenesis and tumor immune tolerance [20]. However, apart from a previous study that identified PAD1 as an EMT that can regulate TNBC and is a biomarker for early oral squamous carcinoma, no studies have been conducted to correlate the tumorigenic potential of PAD1 [12]. Previous studies have always studied single genes as a starting point but ignored the related regulatory role between gene-gene [21, 22]. This study was the first to integrate single genes with their co-expressed genes to develop a CRC risk prediction model for PCGs.

To expand our understanding of the role of PAD1 in CRC, we evaluated the expression of TCGA in colorectal cancer. We showed that PAD1 expression was upregulated in colorectal cancer patients and positively correlated with CRC. We mapped co-expression regulatory networks (PCGs) with PADI1 as the core. We used these co-expressed genes to construct a risk prediction model for CRC. the model constructed by PCGs has the value of predicting CRC prognosis. The constructed Norman diagram can better predict CRC 1, 3, and 5 years survival rates than TNM staging. Moreover, there is a relationship between PCGs and CRC immune microenvironment. Indeed, the immune score was higher in the low-risk group of PCGs, which may be related to the fact that PADI1 has been reported to have an immunosuppressive function, an ability that the tumor may exploit to promote its ability to escape immune cells. The abundance of immune cell infiltration calculated from CIBSORT showed that PADI1 was negatively correlated with CD4+ T cells, plasma cells, and helper T cells and that CD4+ T cell infiltration was less in the low-risk group. In contrast, Tregs cell infiltration was more abundant. Nine genes in the PCGs model correlated with PADI1, so we looked at these genes separately concerning the immune. We, therefore, performed an analysis of the relationship between these genes and immune checkpoints and chemokines separately, which contains both positive and negative correlations, precisely due to the complexity of the PADI1 regulatory network. Finally, we evaluated the relationship between PCGs risk scores and IPS and TIDE. Both immunotherapy predictions suggested better efficacy in the low-risk group than in the high-risk group. This indicates that PADI1 and its co-expressed genes may serve as new markers for clinical immunotherapy and improve clinicians' predictions for CRC immunotherapy. In exploring the mechanisms associated with poor prognosis and poor immunotherapy in the high-risk group of PCGs, we found extracellular matrix remodeling, PPAR signaling pathway, angiogenic signaling pathway, EMT signaling pathway, KRAS signaling pathway, and WNT signaling pathway were highly enriched [23–25]. These pathways were reported to have a relationship with the immune escape of tumors. It is suggested that low-risk patients are immunogenetically “hot” tumors and high-risk patients are immunogenetically “cold” tumors [26].

PAD gene family is all located on the short arm of human chromosome 1, region 3, band 6 (1p36), in a highly clustered gene cluster, hence, the name PADI. Interestingly, this locus is expected to contain a novel, as yet undefined, protein associated with tumorigenesis. In recent years, PADs-mediated protein guanylation has received much attention due to its difference from traditional phosphorylation and acetylation modifications [27]. For example, PADI2 and PADI4 can catalyze the guanylation of histones H3 and H4 at the gene promoter, leading to local alterations in chromatin structure and regulation of tumor-associated gene transcription in human breast cancer cells. Following PADI1-mediated guanylation, the loss of charge on target protein substrates is thought to lead to the breakdown of cytokeratin-polyserin complexes and protein degradation of these target proteins [20]. Apart from its role in epidermal function, we are poorly informed about the potential functions of PADI1 in other physiological or pathological activities.

Despite the merits of the PCGs signature, our study has some limitations that need to be addressed. First, due to the retrospective nature of this study, our views should be interpreted with caution. Second, sampling bias may be unavoidable due to genetic heterogeneity within tumors. Third, although we validated the predictive value of the new signature for prognosis, immune cell infiltration, and treatment response using various methods, external validation is needed for other independent CRC cohorts.

## 5. Conclusions

Genes from PADI1-related co-expression as a newly developed signature show great potential as prognostic biomarkers and immunotherapy predictors in colorectal cancer patients. Prospective studies are essential to further validate the predictive accuracy of this signature before applying it to the individualized management of CRC in a clinical setting.

## Data Availability

Publicly available datasets were used in this study. These data can be found in the cancer genome Atlas (TCGA) database and gene expression omnibus (GEO) database.

## Supplementary Materials

Supplementary Materials Supplementary Figure 1: Distribution and univariate Cox analysis of PADI1 co-expressed genes. Supplementary Figure 2. Construction of the PCGs-based prognostic model. Supplementary Figure 3. Survival analysis and prognostic performance of PCGs signature in GEO cohort for colorectal cancer.
