# Supplementary material for: PADI1 and Its Co-Expressed Gene Signature Unveil Colorectal Cancer Prognosis and Immunotherapy Efficacy
Source: J Oncol. 2022 Nov 26;2022:8394816. doi: 10.1155/2022/8394816 (PMC9719422; doi:10.1155/2022/8394816)
Supplement: Supplementary Materials — Supplementary Figure 1: Distribution and univariate Cox analysis of PADI1 co-expressed genes. Supplementary Figure 2. Construction of the PCGs-based prognostic model. Supplementary Figure 3. Survival analysis and prognostic performance of PCGs signature in GEO cohort for colorectal cancer. [file 8394816.f1.docx]

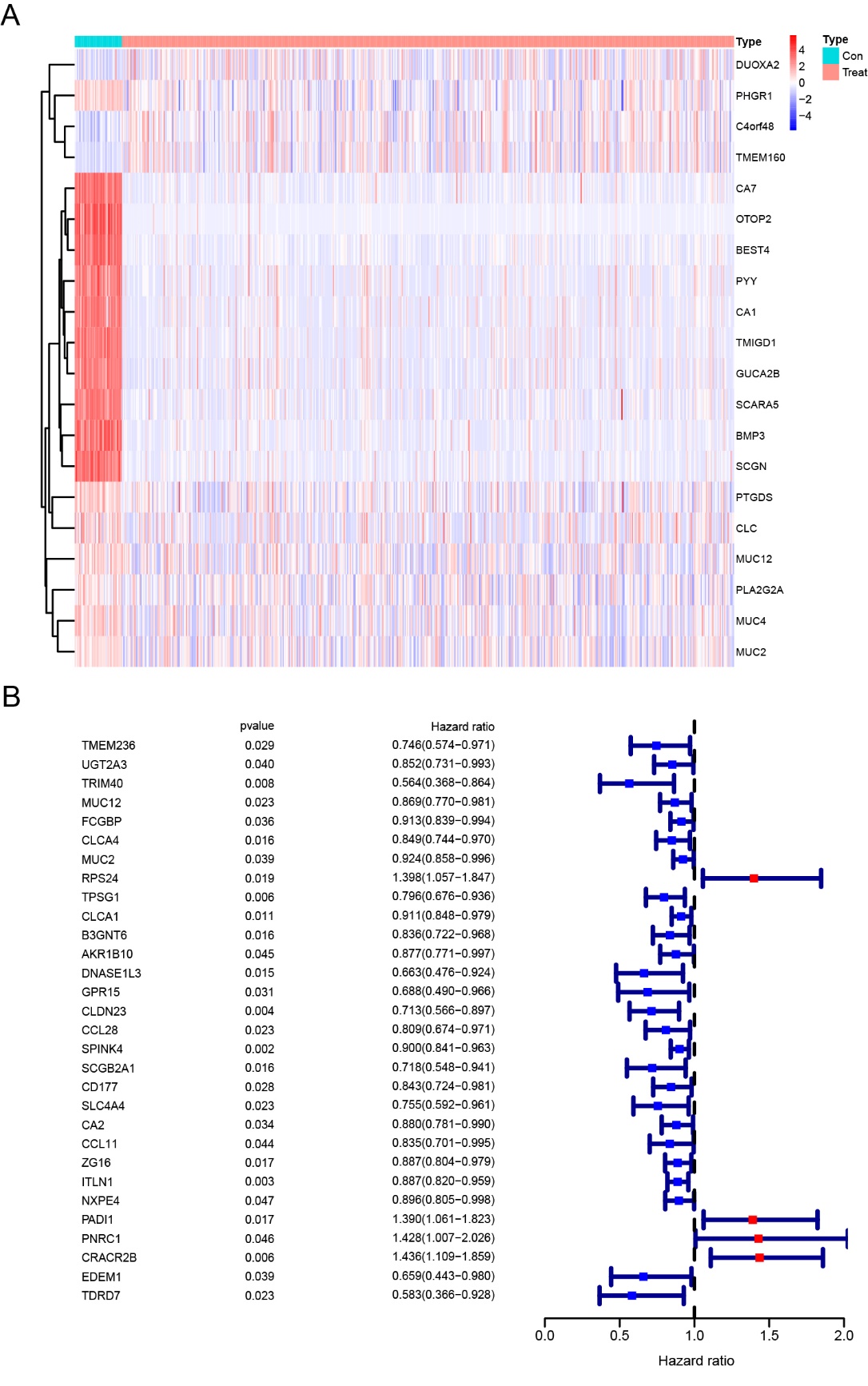


Supplementary Figure 1: Distribution and univariate Cox analysis of PADI1 co-expressed genes. (A) Heatmap of PADI1 co-expressed genes in colorectal cancer versus paracancer. The top 20 genes with the most significant differences are shown.


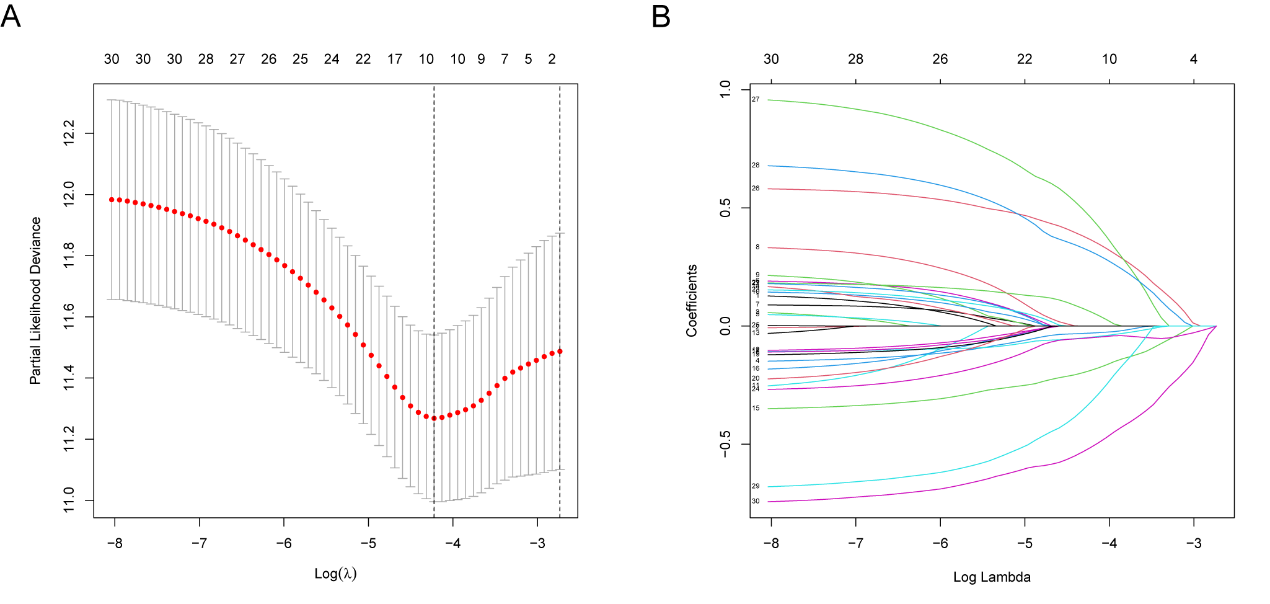


Supplementary Figure 2: Construction of the PCGs-based prognostic model. (A) Cross-validation for tuning the parameter in the LASSO regression. (B) LASSO regression of the 10 OS-related genes.


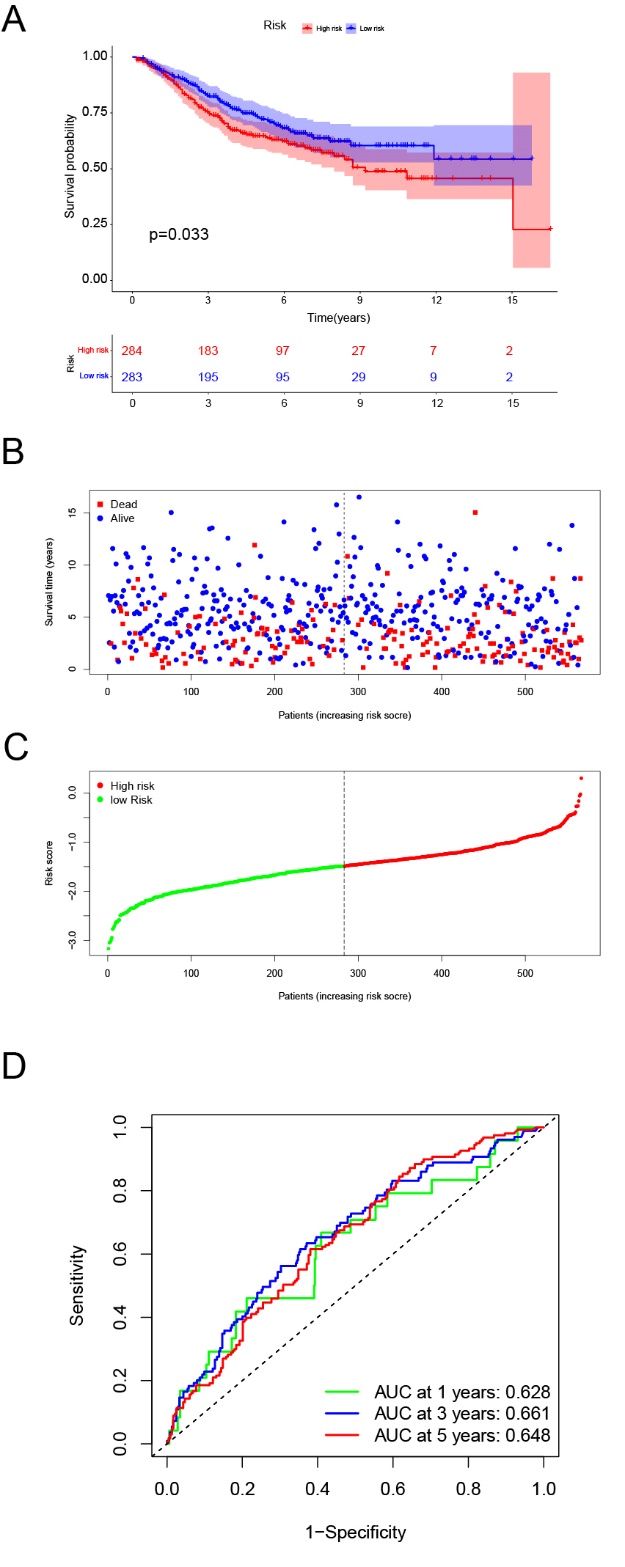


Supplementary Figure 3: Survival analysis and prognostic performance of PCGs signature in GEO cohort for colorectal cancer. (A) Kaplan-Meier analysis for overall survival in colorectal cancer between high- and low-risk patients in the GEO cohort(log-rank test, *p* < 0.05). (B) PCGs risk scores in the GEO cohort of colorectal cancer patients. (C) PCGs survival status in the external GEO cohort of colorectal cancer patients. (D) Receiver operating characteristic (ROC) curve survival at 1/3/5 years in the GEO set predicted by PCGs.
